# Elucidating the etiology of idiopathic spontaneous intraperitoneal hemorrhage

**DOI:** 10.1111/1556-4029.70160

**Published:** 2025-08-25

**Authors:** Dalibor Kovařík, Štěpánka Pohlová Kučerová, Lenka Zátopková, Petr Hejna, Martin Janík

**Affiliations:** ^1^ Department of Forensic Medicine, Faculty of Medicine in Hradec Králové Charles University Hradec Králové Czech Republic; ^2^ Department of Forensic Medicine University Hospital Hradec Králové Hradec Králové Czech Republic; ^3^ Institute of Legal Medicine and Medico‐legal Expertise, Jessenius Faculty of Medicine Comenius University Martin Slovak Republic

**Keywords:** abdominal apoplexy, cirrhosis, ectopic varices, forensic imaging, forensic pathology, idiopathic spontaneous intraperitoneal hemorrhage, nontraumatic hemoperitoneum

## Abstract

Free blood within the abdominal cavity (hemoperitoneum) presents a significant diagnostic and interpretive challenge. It may result from trauma or occur spontaneously in association with underlying disease conditions. When no source of fatal hemorrhage is identified, the implications extend across forensic, criminalistic, legal, and ethical domains. Idiopathic spontaneous intraperitoneal hemorrhage (ISIH), historically known as abdominal apoplexy, is characterized by fatal hemoperitoneum in the absence of trauma or known nontraumatic causes of intraabdominal bleeding. Rupture of a small intraabdominal vessel is suspected; however, the bleeding source usually remains unidentified. We report the case of a 45‐year‐old female with cirrhosis who succumbed to ISIH. Autopsy revealed massive hemoperitoneum and signs of exsanguination. Careful dissection of the abdominal cavity identified hemorrhagic ectopic venous varices beneath the right diaphragmatic arch, covered by an organized blood clot. Histological analysis confirmed recent hemorrhage and demonstrated suspected rupture of the parietal peritoneum near dilated ectopic venous varices. This case is evaluated in the context of pathophysiology, etiological theories, diagnostic limitations, potential contributions of imaging modalities, and forensic relevance. To our knowledge, this is the first reported case of ISIH with dual confirmation—both gross and histological—of hemorrhage originating from ectopic varices. These findings testify that rupture of ectopic venous varices may cause fatal hemorrhage in patients with cirrhosis and underscore the necessity of meticulous autopsy and histopathological correlation.

**Highlights**

- This case presents definitive macroscopic and histologic evidence of the bleeding source in ISIH.
- Ectopic venous varices are confirmed as the bleeding source in a patient with cirrhosis and ISIH.
- Timely recognition and meticulous in situ abdominal examination are critical.
- Extensive histology reinforces macroscopically set source of fatal bleeding.
- Postmortem CT angiography holds potential for identifying the bleeding origin.

## 1 INTRODUCTION

Hemoperitoneum, the presence of free blood in the abdominal cavity, is often a life‐threatening condition with high mortality and diverse clinical presentations depending on the bleeding site and severity [1, 2]. When suspected, diagnostic protocols are promptly initiated to identify the bleeding source; typically, this is followed by surgical, endoscopic, or endovascular interventions to prevent death [1, 3, 4]. From a forensic perspective, detecting hemoperitoneum—whether postmortem CT or autopsy—raises complex questions concerning the cause, source, bleeding rate, contributing factors, and causative nexus. Clarifying these issues falls within the forensic pathologist's scope [5]. Hemoperitoneum may result from trauma or as a complication of disease. This diagnostic challenge parallels that of determining the cause of intracranial hemorrhage or hemothorax [6, 7, 8]. Critically, distinguishing whether the bleeding is of natural or violent origin is a critical decision that shapes the legal investigation.

Idiopathic spontaneous intraperitoneal hemorrhage (ISIH), historically referred to as abdominal apoplexy, is a frequently fatal condition caused by the rupture of a small intraabdominal vessel from natural causes, with an unknown incidence [2, 9, 10, 11, 12]. In forensic practice, autopsies of patients with ISIH often reveal hemoperitoneum and related reactive changes but fail to identify the bleeding source—an omission with serious medicolegal implications [13]. While the literature describes 18 deaths involving patients with ISIH (Table S1) [5, 13, 14, 15, 16, 17, 18, 19, 20], only three published cases provide macroscopic or microscopic photographic evidence of the bleeding origin [16, 17, 18].

Here, we present a case of a patient with ISIH in which the source of fatal intra‐abdominal bleeding was successfully identified both macroscopically and histologically for the first time. The discussion provides a review of reported forensic cases, summarizes current understanding of this enigmatic condition, and offers insights into the diagnostic process. To our knowledge, this is the first case to demonstrate unequivocal macroscopic and microscopic confirmation of the bleeding source in ISIH, testifying that ectopic venous varices may cause fatal intraabdominal hemorrhage in patients with cirrhosis.

## 2 CASE REPORT

A 45‐year‐old female was found lifeless in her apartment. Approximately 1 h before being discovered, she had informed her sister that she was feeling unwell. Upon arrival, the emergency medical team pronounced her dead.

The deceased's medical history (established postautopsy) included advanced liver steatocirrhosis of alcoholic and infectious etiology, complicated by portal hypertension, and multiple activated portocaval anastomoses (clinically identified via abdominal ultrasound). Additional conditions included type II diabetes mellitus, arterial hypertension, mild chronic pancreatitis, episodic epistaxis and hematuria of unknown origin, and a stationary meningioma. She had no history of surgery. A retrospective police investigation found no evidence of blunt abdominal trauma. Two days postmortem, a medical autopsy was performed. No indication for preautopsy imaging was noted.

*External examination* revealed an obese body habitus (BMI = 35.93) and skin jaundice. Healing older hematomas and abrasions were present on the limbs.

**FIGURE 1:**
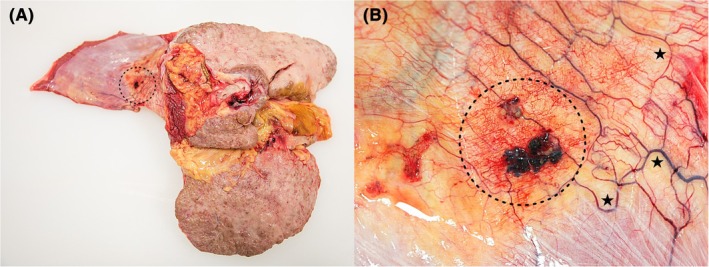
(A) Inferior view of steatocirrhotic liver and peritoneal surface of the right diaphragmatic arch with suspected lesion—dotted circle. (B) Detail showing dilated venous varices surrounded by fresh hemorrhage and covered with a blood clot—dotted circle—along with multiple adjacent dilated ectopic venous varices—asterisks.

*Internal examination* revealed an unexpected finding—massive intraabdominal hemorrhage, with 3 L of liquid and coagulated blood. Layer‐by‐layer dissection of the abdominal wall and thorough in situ evaluation of internal organs revealed no signs of trauma. Moreover, the typical sources of massive abdominal bleeding were absent. Careful inspection of the abdominal cavity revealed distended, congested ectopic varices beneath the parietal peritoneum of the right diaphragmatic arch, accompanied by significant bleeding and a small blood clot around (Figure 1). Dissection of internal organs confirmed severe liver steatocirrhosis (1820 g), yellow discoloration of mucosal and soft tissues, splenomegaly (440 g), and cardiac hypertrophy (590 g). Esophageal varices and adhesions were not observed.

**FIGURE 2:**
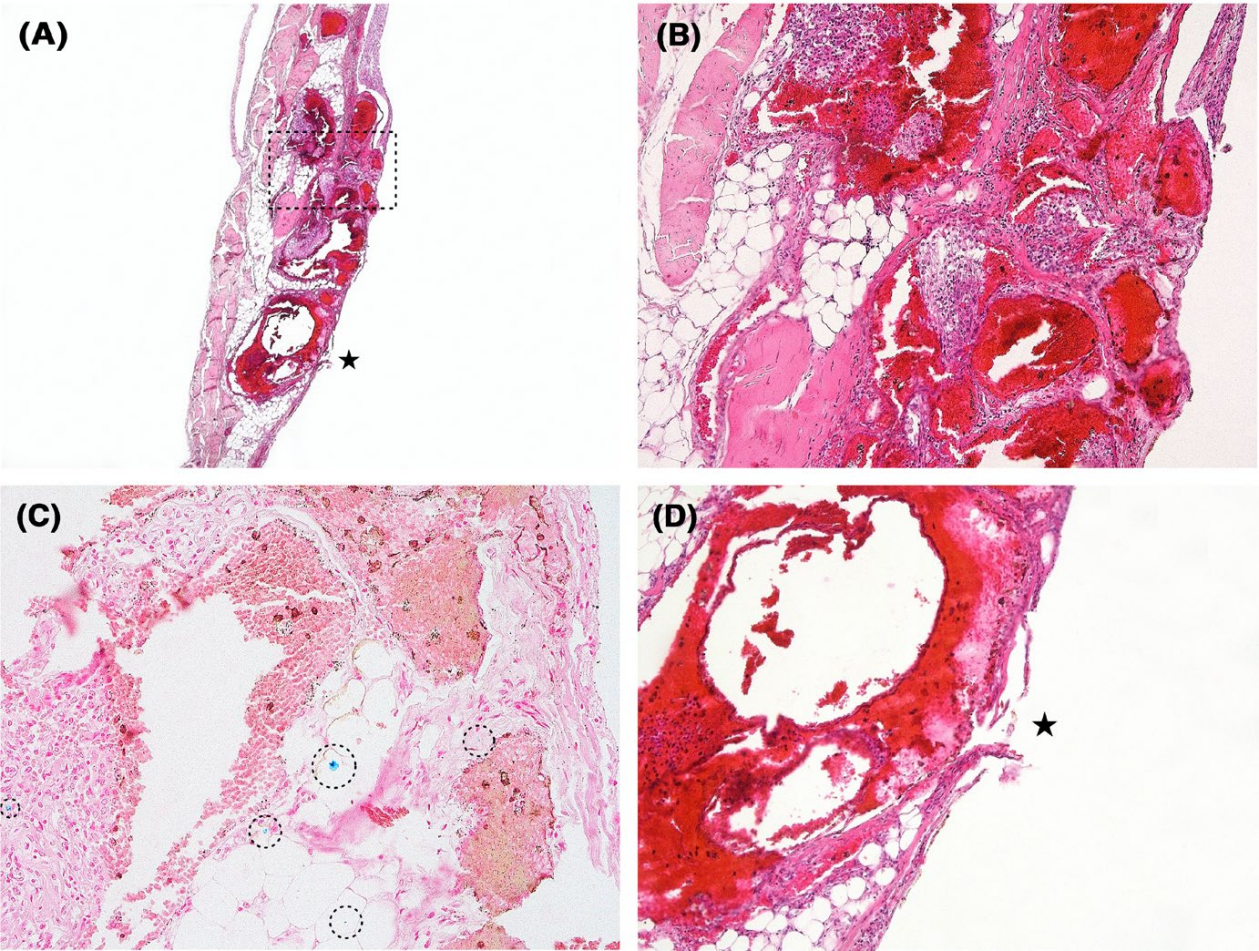
Histological examination of the suspected lesion on the parietal portion of the right diaphragmatic arch. (A) Numerous congested, dilated ectopic varices, rupture of the parietal peritoneum—asterisk—and an area showing characteristic chronic changes—dotted rectangle (H&E; 2×). (B) Detail of chronic inflammatory infiltration and fibroproductive changes in surrounding soft tissue within the area marked by the dotted rectangle (H&E; 10×). (C) Hemosiderin deposits within soft tissue—dotted circles (Perl's Prussian blue; 20×). (D) Suspected rupture of the parietal peritoneum with surrounding fresh hemorrhage—asterisk (H&E; 10×).

Histological examination of nearly 100 tissue samples from the suspected area revealed numerous thick‐walled, congested, and enlarged varices in close proximity, exhibiting fresh bleeding into adjacent soft tissue and mixed inflammatory infiltration (Figure 2A,B). Perl's Prussian blue staining confirmed hemosiderin deposits (Figure 2C). Definitive analysis showed a suspected rupture of the parietal peritoneum near the dilated ectopic varices and hemorrhaged soft tissue (Figure 2D).

Toxicological analysis detected trace amounts of mirtazapine, sertraline, and carvedilol in the blood. The cause of death was determined to be fatal intraabdominal hemorrhage due to rupture of ectopic venous varices associated with advanced portal hypertension. The manner of death was classified as natural.

## 3 DISCUSSION

Distinguishing between traumatic and atraumatic hemoperitoneum presents a significant diagnostic challenge for forensic pathologists, although identifying the source of fatal hemorrhage during autopsy is typically straightforward. Traumatic causes include penetrating or blunt force injuries to the torso that are usually connected with skin or internal organ injuries. Iatrogenic hemoperitoneum, resulting from complications during diagnostic or therapeutic procedures, could be a distinct traumatic subtype [21, 22, 23, 24, 25, 26]. Conversely, *spontaneous hemoperitoneum* can result from various natural causes, including rupture of large vessels, cyst rupture, vascular malformations, tumor complications, gynecologic and obstetric conditions, acute hemorrhagic pancreatitis, splenic infections, idiopathic omental hemorrhage, and coagulopathies [1, 3, 4, 16, 27, 28, 29, 30, 31, 32, 33]. Notably, in nearly 30% of spontaneous hemoperitoneum cases, the source of bleeding remains undetermined despite thorough examination [13, 16, 20, 34].

ISIH refers to spontaneous intraabdominal bleeding, typically fatal, with no identifiable cause following exclusion of trauma and other known etiologies [2, 9, 10, 11, 12]. ISIH also includes cases where the exact bleeding source remains elusive despite thorough investigation. This condition is more frequently reported in males (male:female ratio 2–3:1), predominantly in individuals aged 40–60 years [2, 16, 18]. The first case was described by Barber in 1909 in a young female during childbirth when laparotomy failed to identify the hemorrhage source [35]. In 1931, Green and Power introduced the term “abdominal apoplexy” while reporting five cases; in one, the bleeding source remained undetected [36].

Although ISIH is labeled as “spontaneous,” it is incorrect to assume that intraabdominal bleeding occurs without cause or always involves massive hemorrhage [5, 37]. Frequently, underlying vascular lesions remain undetected during autopsy, even when present [5, 16]. The source of fatal bleeding in ISIH may be either arterial or venous. Arterial sources often involve aneurysms of branches of the abdominal aorta [16], with variable localization rates: splenic artery (60%), renal artery (22%), hepatic artery (10–20%), mesenteric artery (5.5%), celiac and gastrointestinal/epiploic arteries (4%), intestinal artery (3%), and duodenal/pancreatic artery (1.5%) [10, 11, 18, 20, 38]. Possible substrates for arterial rupture could be inflammatory vascular damage, congenital connective tissue disorders, and chronic drug use—particularly cocaine [8, 9, 10, 16, 36, 39, 40]. The second major subcategory of ISIH cases involves fatalities among individuals with severe liver pathology and advanced portal hypertension. Portal hypertension, typically resulting from liver cirrhosis, is closely associated with various complications and reactive systemic changes. A hallmark of advanced portal hypertension is the activation of portocaval anastomoses, for example, esophageal varices, caput medusae, hemorrhoids [41]. In addition, small ectopic venous varices, distributed throughout the abdominal cavity‘s soft tissues and within internal organs beyond the esophagogastric region, may also become engorged [4, 5, 17, 39, 40, 42, 43]. Ectopic varices are usually found in the duodenum, jejunum, ileum, colon, and rectum [4, 17, 43, 44, 45, 46]. Less frequent sites include the ovary, vagina, and peritoneum—specifically the right diaphragmatic area in some reports—with the latter having an incidence of 9% [43, 45, 47]. Some authors have proposed that rupture of ectopic venous varices in patients with cirrhosis and portal hypertension may be a likely cause of fatal intra‐abdominal hemorrhage in ISIH [5, 17]. Aside from portal hypertension, ectopic venous varices may arise due to congenital portosystemic anastomoses, abnormal vascular architecture, systemic vasculitis, or arteriovenous fistulae [43].

The susceptibility of ectopic venous varices to rupture is primarily due to their diminutive size and fragile structure. Provocative events lead to sudden increase in intraabdominal pressure and include lifting heavy objects, intense vomiting, forceful coughing, or defecation [28, 29, 39, 48]. Although ISIH is defined as “nontraumatic,” minor mechanical stress or borderline physical exertion—such as torsional movements, sudden jerks, or a fall onto the buttocks—may still provoke vascular rupture. Such events would typically be inconsequential in healthy individuals.

The pathophysiology of ISIH is multifactorial. As with other hemorrhagic conditions, acute illnesses and underlying disorders affecting coagulation substantially impair hemostatic capacity and exacerbate bleeding. In addition to the activation of ectopic varices, severe hepatic dysfunction reduces the synthesis of coagulation factors [5, 17]. Acute alcohol intake may further compromise hemostasis by causing thrombocytopenia, prolonging clotting time, and inhibiting collagen‐induced platelet aggregation [5, 13, 14, 49]. A potentiating effect is likely when acute alcohol use occurs alongside chronic hepatic damage [13]. Other medical conditions (such as myeloma, polycythemia vera, hemophilia, or congenital factor X deficiency) as well as anticoagulant therapy may also severely impair coagulation [50, 51, 52, 53]. As previously mentioned, arterial hypertension plays a crucial role in the pathophysiology and should be considered an additional contributor to the rupture of compromised small vessels [9, 19, 54, 55, 56]. Other comorbidities and advanced age may indirectly worsen overall health and diminish the body's capacity to respond effectively to hemorrhage. Last, it is important to consider the cumulative effect of all factors adversely affecting coagulation.

A review of the literature identified only 18 cases of ISIH (Table S1). Among the 18 forensic cases identified, eight involved male individuals and nine females; in one case, sex was not specified. Ten individuals were aged 30–50 years, six were aged 50–70 years, and one was aged ≥70 years; age was not reported in one case. Severe liver disease—specifically cirrhosis—was documented in 14 cases, including six with alcoholic etiology. Clinical or laboratory signs of coagulation disorders were noted in only three cases. Alcohol was present at the time of death in three individuals, and three tested positive for other substances, including one with toxic levels of alprazolam. In four cases, additional chronic natural pathological findings were identified. Of all reported forensic cases, 12 did not determine the source of fatal hemorrhage during autopsy. In five cases, autopsy and histology identified the bleeding source; however, only three publications provided conclusive evidence—Hayshi included a macroscopic image, and Harbour and Podduturi each included microscopic images [16, 17, 18].

Harbour et al. reported two individuals who died from hypovolemic shock due to dissection of the gastroduodenal artery and pseudoaneurysm at the junction of the superior mesenteric and portal veins, one of them confirmed by histology [16]. Hayshi presented two fatal cases of exsanguination from ectopic varices within the mesentery of the ascending colon and varices of the hernial sac of a congenital left inguinal hernia, both evidenced by macroscopic images [17]. Another case involved a 59‐year‐old female with histology confirming rupture of a dissected gastroduodenal artery likely associated with mycotic vasculitis [18].

More than a decade after the last published ISIH case with an identified bleeding source, we present a case with several ISIH features and a confirmed origin of fatal intraabdominal hemorrhage. Police investigation and autopsy excluded prior blunt trauma. The deceased, who had long‐standing hepatic steatocirrhosis and portal hypertension, was found to have radiologically confirmed portocaval anastomoses within the abdominal cavity—findings unknown to forensic pathologists until postmortem examination. Unlike in Hayshi's case, the decedent had no history of abdominal surgery, and the bleeding site at the right diaphragmatic arch was unrelated to postoperative adhesions [17]. The right diaphragmatic arch is an uncommon hemorrhage site [43, 45, 47]. The deceased's medical history included likely signs of coagulation dysfunction, such as prior episodes of epistaxis and hematuria—features consistent with previously reported ISIH cases. Additional comorbidities—including type II diabetes mellitus, arterial hypertension, and mild chronic pancreatitis—may have compromised physiological resilience and contributed to the fatal outcome. Toxicology testing detected no alcohol; trace levels of mirtazapine, sertraline, and carvedilol were present but were not considered to have impacted hemostasis.

Macroscopic detection of the source of fatal bleeding in ISIH remains a complex and often unsuccessful task for forensic pathologists, despite early suspicion and correct autopsy technique—meticulous evacuation of abdominal contents and thorough in situ examination of the abdominal cavity prior to organ evisceration under optimal lighting conditions. Adequate surgical lighting enhances visual acuity, while a suction unit can aid in the careful removal of abdominal contents for subsequent visual, cytological, or histological analysis to determine the hemorrhage's origin (e.g., tumorous, pyogenic, or reactive). Use of magnifying lenses can further enhance precision. Exclusive reliance on organ dissection may be limiting, as standard evisceration can alter or obscure crucial structures such as the diaphragm, abdominal wall, pelvic floor, and mesenteric attachments. This may lead to missed vessel ruptures or the introduction of artifacts, ultimately misguiding the diagnostic process. To minimize artifact formation, less commonly used evisceration techniques, such as the Ghon (*en bloc*) and Letulle (*en masse*) methods, may better preserve anatomical integrity and spatial relationships. Thorough examination of the abdominal cavity and subsequent evisceration must always be performed strictly under the supervision of a forensic pathologist. Nonetheless, identifying the bleeding source may fail despite best efforts, due to several variables. Extensive soft tissue hemorrhage may either guide the examiner's focus or obscure a minor bleeding source. Furthermore, the morphology of ectopic venous varices—including their small size, atypical locations, and tendency to collapse post‐rupture, along with minimal or absent vascular filling after exsanguination—significantly complicates their detection [18]. Postmortem changes may further alter soft tissue findings.

Extensive histological examination of suspicious regions could strengthen and confirm macroscopically estimated sources of fatal bleeding. Chronic inflammatory infiltrates, reactive fibrosis, and edema in tissues surrounding ectopic venous varices may indicate long‐term vascular activation [57, 58, 59]. Reactive fibrosis may offer limited protection against rupture, while hemosiderin deposits near varices suggest prior episodic bleeding [60]. Fresh hemorrhage along ectopic venous varices may signal ongoing bleeding [60]. Rupture of the vessel wall may be confirmed by the definitive histological finding, despite the potential for artifacts to be formed during histological processing.

In clinical settings, imaging methods play a central role in detecting life‐threatening bleeding, complementing exploratory laparotomy and endovascular procedures [1, 2, 3, 9, 10, 12, 13, 18, 32, 33, 34, 38, 43, 56, 61]. Similarly, postmortem imaging methods could be helpful in fatal ISIH cases. Postmortem CT scanning can confirm intraabdominal blood accumulation; however, it often fails to identify the bleeding source [62]. Postmortem CT angiography, though still largely experimental [63], offers accurate identification of bleeding sources in deceased individuals [63, 64, 65]. To date, no published ISIH cases have confirmed the bleeding source via postmortem CT angiography. However, with early anticipation, timely indication, and appropriate application, this technique could become diagnostically equivalent to the dual standard of macroscopic and histologic confirmation.

## 4 CONCLUSION

In summary, ISIH may pose significant forensic challenges. Early anticipation is critical for the successful detection of atypically located bleeding sources and requires the forensic pathologist's attention, diligence, and perseverance. A second pivotal factor is histological confirmation of the bleeding source. Looking ahead, preautopsy whole‐body CT angiography holds promise as a diagnostic modality capable of localizing fatal bleeding sources and serving as an equivalent to conventional methods. Despite optimal procedures, the bleeding source may remain undetected in some cases. This case provides a foundation for a proposal to The Czech Society for Legal Medicine and Forensic Toxicology to develop autopsy and diagnostic guidelines for cases of fatal hemoperitoneum.

## CONFLICT OF INTEREST STATEMENT

The authors have no conflicts of interest to declare.

## Supporting information


**Table S1.** Main features of all available forensic cases of ISIH. ^†^Case with failure to retain the autopsy report; ^‡^Case with incomplete description. BAC, blood alcohol content; DIC, disseminated intravascular coagulation; F, female; M, male; Ma, macroscopic image; Mi, microscopic image.

## Data Availability

Data sharing is not applicable to this article as no new data were created or analyzed in this study.

## References

[1] Zhou Y , Zhou Y , Li W , Lin S . Idiopathic spontaneous intraperitoneal hemorrhage due to vascular malformations in the muscularis of the stomach: A case report. Front Med. 2022;9:927899. 10.3389/fmed.2022.927899 PMC9474647 36117972

[2] McComb K , Barghash M , Eltayef S . Idiopathic spontaneous intraperitoneal haemorrhage (ISIH): A diagnostic dilemma and its conservative management. Cureus. 2023;15(9):e44879. 10.7759/cureus.44879 37814746 PMC10560380

[3] Lucey BC , Varghese JC , Anderson SW , Soto JA . Spontaneous hemoperitoneum: A bloody mess. Emerg Radiol. 2007;14(2):65–75. 10.1007/s10140-007-0594-0 17342463

[4] Aseni P , Di Domenico SL , Barbosa F , Rampoldi A , Berry C . Hemoperitoneum in cirrhotic patients in the absence of abdominal trauma. Expert Rev Gastroenterol Hepatol. 2019;13(9):867–876. 10.1080/17474124.2019.1631159 31204541

[5] Kovarik D , Hejna P . Comments on death due to non‐traumatic hemoperitoneum in Milan 2002–2016, with focus on two cases of abdominal apoplexy (idiopathic spontaneous hemoperitoneum) and review of the literature. Leg Med. 2017;31:30–32. 10.1016/j.legalmed.2017.12.006 29275287

[6] Quiñones‐Ossa GA , Durango‐Espinosa Y , Padilla‐Zambrano H , Moscote‐Salazar LR , Keni R , Deora H , et al. The puzzle of spontaneous versus traumatic intracranial hemorrhages. Egypt J Neurosurg. 2020;35(1):13. 10.1186/s41984-020-00084-9

[7] Vrbanic L , Hunt C , Cooney M , Heffernan J , Walsh A , Heaney C , et al. Intracranial haemorrhage and falls: Cause or effect? Ir J Med Sci. 2023;192(5):2387–2390. 10.1007/s11845-022-03222-4 36534315

[8] Janik M , Straka L , Krajcovic J , Hejna P , Hamzik J , Novomesky F . Non‐traumatic and spontaneous hemothorax in the setting of forensic medical examination: A systematic literature survey. Forensic Sci Int. 2014;236:22–29. 10.1016/j.forsciint.2013.12.013 24529771

[9] Wang H , Xiu D . Abdominal apoplexy because of the rupture of gastroduodenal artery and inferior pancreaticoduodenal artery: A case report. Medicine (Baltimore). 2017;96(43):e8264. 10.1097/MD.0000000000008264 29068993 PMC5671826

[10] Qaraqe TM , Abou Daher A , Alami RS . Abdominal apoplexy: A rare case of spontaneous middle colic artery rupture with transverse colectomy. Int J Surg Case Rep. 2021;81:105835. 10.1016/j.ijscr.2021.105835 33887831 PMC8027271

[11] Negmadjanov U , Ohanisian L , Rubay D , Hristov B , Belizon A . Abdominal apoplexy: A case study of idiopathic spontaneous lesser sac hematoma. Cureus. 2019;11(6):e4937. 10.7759/cureus.4937 31431842 PMC6695238

[12] Badri F , Packirisamy K , Aryasinghe L , Al Suwaidi M . Abdominal apoplexy: A rare case of spontaneous rupture of the superior mesenteric artery in a hypertensive patient. Int J Surg Case Rep. 2012;3(12):614–617. 10.1016/j.ijscr.2012.07.013 23017492 PMC3484817

[13] Dedouit F , Piercecchi‐Marti MD , Leonetti G , Rougé D , Telmon N . Unexpected natural death secondary to intra‐abdominal bleeding: Report of one idiopathic spontaneous intraperitoneal hemorrhage case. Forensic Sci Int. 2012;214(1–3):e43–e46. 10.1016/j.forsciint.2011.08.004 21872410

[14] Di Maio VJ . Sudden, unexpected death due to massive, nontraumatic intra‐abdominal hemorrhage in association with cirrhosis of the liver. Am J Forensic Med Pathol. 1987;8(3):266–268. 10.1097/00000433-198708030-00015 3673990

[15] Kim HJ , Kim YH , Chung NE , Seo JS . Unexpected death due to massive intra‐abdominal hemorrhage in association with liver cirrhosis. Korean J Leg Med. 2007;31:92–94.

[16] Harbour LN , Koch MS , Louis TH , Fulmer JM , Guileyardo JM . Abdominal apoplexy: Two unusual cases of hemoperitoneum. Proc (Bayl Univ Med Cent). 2012;25(1):16–19. 10.1080/08998280.2012.11928772 22275776 PMC3246846

[17] Hayashi T , Buschmann C , Matejic D , Ingold B , Tsokos M . Fatal intra‐abdominal bleeding from ectopic varices: Report of two autopsy cases. Forensic Sci Med Pathol. 2013;9(1):96–99. 10.1007/s12024-012-9326-1 22383173

[18] Podduturi V , Guileyardo JM . Abdominal apoplexy: A stroke of misfortune. Acad Forensic Pathol. 2014;4(1):118–122. 10.23907/2014.020

[19] Battistini A , Marchesi M , Amadasi A , Rancati A , Gentile G , Zoja R . Death due to non‐traumatic hemoperitoneum in Milan 2002–2016, with focus on two cases of abdominal apoplexy (idiopathic spontaneous hemoperitoneum) and review of the literature. Leg Med (Tokyo). 2017;29:13–17. 10.1016/j.legalmed.2017.09.003 28964983

[20] Mileva B , Georgieva M , Tsranchev II , Goshev M , Gulinac M , Alexandrov A . Abdominal apoplexy: Sudden death due to massive, nontraumatic intra‐abdominal hemorrhage. Cureus. 2023;15(10):e47335. 10.7759/cureus.47335 38021727 PMC10657205

[21] Greenberg A , Bernardini J , Piraino BM , Johnston JR , Perlmutter JA . Hemoperitoneum complicating chronic peritoneal dialysis: Single‐center experience and literature review. Am J Kidney Dis. 1992;19(3):252–256. 10.1016/s0272-6386(13)80006-6 1553970

[22] De Santis G , Gola P , Lancione L , Sista F , Pietroletti R , Leardi S . Sigmoid intramural hematoma and hemoperitoneum: An early severe complication after stapled hemorrhoidopexy. Tech Coloproctol. 2012;16(4):315–317. 10.1007/s10151-011-0696-2 21678070

[23] Andreuccetti J , Gaj F , Crispino P , Dassatti MR , Negro P . Hemoperitoneum: A rare complication of hemorrhoid treatment. Tech Coloproctol. 2014;18(4):399–401. 10.1007/s10151-012-0849-y 22706732

[24] Zhu Z , Yin J , Zhu H , Cao H . An unusual case of life‐threatening hemoperitoneum after colonoscopy. Endoscopy. 2015;47(Suppl 1):E468–E469. 10.1055/s-0034-1392972 26465188

[25] Mercky P , Gonzalez JM , Caillol F , Bories E , Pesenti C , Giovannini M . Hemoperitoneum after endoscopic mucosal resection for Barrett's esophagus. Endoscopy. 2014;46(Suppl 1):E15. 10.1055/s-0033-1359161 24446097

[26] Buch KE , Reiner M , Divino CM . Hemoperitoneum following inguinal hernia repair: A case report. Hernia. 2007;11(5):459–461. 10.1007/s10029-007-0212-9 17332970

[27] Zeinalpour A , Aghili A , Gholizadeh B . Abdominal apoplexy due to rupture of inferior pancreaticoduodenal artery: A rare case of acute abdomen. Caspian J Intern Med. 2021;12(Suppl 2):S479–S481. 10.22088/cjim.12.0.479 34760110 PMC8559655

[28] Yao F , Ding H , Yan H , Zhang F , Wang N . Spontaneous hemoperitoneum in pregnancy. Taiwan J Obstet Gynecol. 2021;60(4):796–797. 10.1016/j.tjog.2021.05.041 34247831

[29] Zhang Y , Zhao Y , Wei Y , Li R , Qiao J . Spontaneous rupture of subserous uterine veins during late pregnancy after in vitro fertilization. Fertil Steril. 2009;92(1):395.e13–395.e16. 10.1016/j.fertnstert.2009.03.096 19463999

[30] Schwartz M , Powell K . Spontaneous rupture of a leiomyoma causing life‐threatening intra‐abdominal hemorrhage. Case Rep Obstet Gynecol. 2017;2017:3701450. 10.1155/2017/3701450 28127487 PMC5239864

[31] McDowell E , Hughes K , Jones M , Kimpson C , Twiner MJ , McCormick S . Hidden hemorrhage: A case of idiopathic omental hemorrhage causing spontaneous hemoperitoneum. J Am Coll Emerg Physicians Open. 2024;5(4):e13242. 10.1002/emp2.13242 39027349 PMC11255013

[32] Lovitskyi Y , Romanenko Y , Shcherbyna M , Zadorozhna K , Kalyna R , Herasymenko E , et al. Unusual spontaneous intraperitoneal hemorrhage: Three case reports. J Surg Case Rep. 2024;2024(8):rjae475. 10.1093/jscr/rjae475 39109380 PMC11298317

[33] Ballesta M , Piqueras R , Brugger S , Estellés Lerga P . Non‐traumatic spontaneous abdominal haemorrhage. Radiologia (Engl Ed). 2023;65(Suppl 1):S73–S80. 10.1016/j.rxeng.2022.11.004 37024233

[34] Law EKC , Lee RKL , Hung EHY , Ng AWH . Radiological diagnosis and management of idiopathic spontaneous intra‐abdominal haemorrhage (abdominal apoplexy): A case series. Abdom Imaging. 2015;40(2):343–351. 10.1007/s00261-014-0220-z 25134802

[35] Barber MC . Intra‐abdominal haemorrhage associated with labour. Br Med J. 1909;2(2534):203–204. 10.1136/bmj.2.2534.203-a PMC2320277 20764605

[36] Green WT , Powers JH . Intra‐abdominal apoplexy. Ann Surg. 1931;93(5):1070–1074. 10.1097/00000658-193105000-00013 17866563 PMC1398357

[37] Carter R , Gosney WG . Abdominal apoplexy. Report of six cases and review of the literature. Am J Surg. 1966;111(3):388–397. 10.1016/s0002-9610(66)80017-x 5907006

[38] Jadav M , Ducheine Y , Brief D , Carter L , McWhite T , Hardy J . Abdominal apoplexy: A case study of the spontaneous rupture of the gastroepiploic artery. Curr Surg. 2004;61(4):370–372. 10.1016/j.cursur.2004.01.005 15276342

[39] Ma YJ , Chen EQ , Lu JJ , Tan MZ , Tang H . Hemoperitoneum in cirrhotic patients without abdominal trauma or tumor. Hepatobiliary Pancreat Dis Int. 2011;10(6):644–648. 10.1016/s1499-3872(11)60109-4 22146630

[40] Chu EC , Chick W , Hillebrand DJ , Hu KQ . Fatal spontaneous gallbladder variceal bleeding in a patient with alcoholic cirrhosis. Dig Dis Sci. 2002;47(12):2682–2685. 10.1023/a:1021092719209 12498285

[41] Sincos IR , Mulatti G , Mulatti S , Sincos IC , Belczak SQ , Zamboni V . Hemoperitoneum in a cirrhotic patient due to rupture of retroperitoneal varix. HPB. 2009;2009:240780. 10.1155/2009/240780 19404409 PMC2673472

[42] Matsui M , Kojima A , Kakizaki S , Nagasaka K , Sohara N , Sato K , et al. Ectopic varices in a right diaphragm that ruptured into the pleural cavity. Acta Med Okayama. 2006;60(4):229–232. 10.18926/AMO/30716 16943860

[43] Akhter NM , Haskal ZJ . Diagnosis and management of ectopic varices. Gastrointest Interv. 2012;1(1):3–10. 10.1016/j.gii.2012.08.0010

[44] House T , Webb P , Baarson C . Massive hemorrhage from ectopic duodenal varices: Importance of a multidisciplinary approach. Case Rep Gastroenterol. 2017;11(1):36–41. 10.1159/000455184 28203136 PMC5301088

[45] Sato T , Akaike J , Toyota J , Karino Y , Ohmura T . Clinicopathological features and treatment of ectopic varices with portal hypertension. Int J Hepatol. 2011;2011:960720. 10.4061/2011/960720 21994879 PMC3170857

[46] Almadi MA , Almessabi A , Wong P , Ghali PM , Barkun A . Ectopic varices. Gastrointest Endosc. 2011;74(2):380–388. 10.1016/j.gie.2011.03.1177 21612777

[47] Helmy A , Al Kahtani K , Al Fadda M . Updates in the pathogenesis, diagnosis and management of ectopic varices. Hepatol Int. 2008;2(3):322–334. 10.1007/s12072-008-9074-1 19669261 PMC2716887

[48] Wu CY , Hwang JL , Lin YH , Hsieh BC , Seow KM , Huang LW . Spontaneous hemoperitoneum in pregnancy from a ruptured superficial uterine vessel. Taiwan J Obstet Gynecol. 2007;46(1):77–80. 10.1016/S1028-4559(08)60114-X 17389197

[49] Tsokos M , Türk EE . Esophageal variceal hemorrhage presenting as sudden death in outpatients. Arch Pathol Lab Med. 2002;126(10):1197–1200. 10.5858/2002-126-1197-EVHPAS 12296758

[50] Lucey BC , Varghese JC , Soto JA . Spontaneous hemoperitoneum: Causes and significance. Curr Probl Diagn Radiol. 2005;34(5):182–195. 10.1067/j.cpradiol.2005.06.001 16129236

[51] Estivill Pallejà X , Domingo P , Fontcuberta J , Félez J . Spontaneous retroperitoneal hemorrhage during oral anticoagulant therapy. Arch Intern Med. 1985;145(8):1531, 1534. 10.1001/archinte.1985.00360080213039 3161482

[52] Xiao X , Zhu W , Dai Q . Direct oral anticoagulants versus traditional anticoagulation in cirrhotic patients with portal vein thrombosis: Updated systematic review. Clin Appl Thromb Hemost. 2024;30:10760296241303758. 10.1177/10760296241303758 39587933 PMC11590147

[53] Prince S , Dayto DC , Sephien A , Lozano M , Tobillo R , Hurlock NP , et al. Efficacy and safety of direct oral anticoagulants compared to warfarin in patients with cirrhosis and splanchnic vein thrombosis. South Med J. 2024;117(11):662–665. 10.14423/SMJ.0000000000001750 39486452

[54] Kleinsasser LJ . Abdominal apoplexy. Report of two cases and review of the literature. Am J Surg. 1970;120(5):623–628. 10.1016/s0002-9610(70)80181-7 5313966

[55] Aldhafeeri S , Aljoaib A , Alghumlas S , Alotaibi F , Alghamdi R , Alghazwi A . A rare cause of acute abdomen in middle‐aged man: Idiopathic spontaneous hemoperitoneum. Int J Surg Case Rep. 2022;99:107691. 10.1016/j.ijscr.2022.107691 36152370 PMC9568867

[56] Badri D , Killoran C , Aseervatham R . Idiopathic spontaneous intraperitoneal haemorrhage: A near fatal presentation of acute abdomen requiring prompt diagnosis. Int J Surg Case Rep. 2023;110:108650. 10.1016/j.ijscr.2023.108650 37603915 PMC10445459

[57] Song E , Ouyang N , Hörbelt M , Antus B , Wang M , Exton MS . Influence of alternatively and classically activated macrophages on fibrogenic activities of human fibroblasts. Cell Immunol. 2000;204(1):19–28. 10.1006/cimm.2000.1687 11006014

[58] Wynn TA , Barron L . Macrophages: Master regulators of inflammation and fibrosis. Semin Liver Dis. 2010;30(3):245–257. 10.1055/s-0030-1255354 20665377 PMC2924662

[59] Wynn TA , Ramalingam TR . Mechanisms of fibrosis: Therapeutic translation for fibrotic disease. Nat Med. 2012;18(7):1028–1040. 10.1038/nm.2807 22772564 PMC3405917

[60] Dettmeyer R . Vitality, injury age, determination of skin wound age, and fracture age. In: Dettmeyer R , editor. Forensic histopathology: Fundamentals and perspectives. Heidelberg, Germany: Springer; 2011. p. 195.

[61] Saeed Y , Farkas Z , Azeez S . Idiopathic spontaneous intraperitoneal hemorrhage due to rupture of short gastric artery presenting as massive gastrointestinal bleeding: A rare case presentation and literature review. Cureus. 2020;12(11):e11499. 10.7759/cureus.11499 33354445 PMC7744231

[62] Tsaklakidis A . Imaging methods. In: Dedouit F , Yen K , Heinze S , editors. Forensic imaging: A practical guide. Cham, Switzerland: Springer International Publishing; 2022. p. 15–16.

[63] Grabherr S , Grimm J , Dominguez A , Vanhaebost J , Mangin P . Advances in post‐mortem CT‐angiography. Br J Radiol. 2014;87(1036):20130488. 10.1259/bjr.20130488 24234582 PMC4067028

[64] Jackowski C , Sonnenschein M , Thali MJ , Aghayev E , von Allmen G , Yen K , et al. Virtopsy: Postmortem minimally invasive angiography using cross section techniques—Implementation and preliminary results. J Forensic Sci. 2005;50(5):1175–1186. 10.1520/JFS2005023 16225226

[65] Grabherr S , Doenz F , Steger B , Dirnhofer R , Dominguez A , Sollberger B , et al. Multi‐phase post‐mortem CT angiography: Development of a standardized protocol. Int J Legal Med. 2011;125(6):791–802. 10.1007/s00414-010-0526-5 21057803

