# Supplementary material for: Elucidating the etiology of idiopathic spontaneous intraperitoneal hemorrhage
Source: J Forensic Sci. 2025 Aug 25;70(6):2565–71. doi: 10.1111/1556-4029.70160 (PMC12584118; doi:10.1111/1556-4029.70160)
Supplement: Supplementary file 1 — Table S1. Main features of all available forensic cases of ISIH. †Case with failure to retain the autopsy report; ‡Case with incomplete description. BAC, blood alcohol content; DIC, disseminated intravascular coagulation; F, female; M, male; Ma, macroscopic image; Mi, microscopic image. [file JFO-70-2565-s001.docx]

| **first author/**  **year of publication (reference)** | **gender/age** | **place of death/activity** | **complaints/**  **clinical features** | **liver impairment/**  **etiology** | **comorbidities** | **coagulopathy** | **blood volume in abdominal cavity** | **liver/**  **spleen/**  **heart**  **weight (g)** | **BAC** | **toxicology** | **source of bleeding** | **form of proof** |
| --- | --- | --- | --- | --- | --- | --- | --- | --- | --- | --- | --- | --- |
| DiMaio/  1987 [14] | F/44 y | home/sitting | none | micronodular cirrhosis, with fatty metamorphosis /  ethylic | none | „probably“ | 2750 ml  nonclotted blood | 1880/  115/  240 | negative | negative | unknown | --- |
| DiMaio/  1987 [14] | M/38 y | street/walking | none | micronodular cirrhosis, with fatty metamorphosis /  ethylic | none | „probably“ | 4800 ml nonclotted blood | 2110/  510/  510 | negative | not mentioned | unknown | --- |
| DiMaio/  1987 [14] | M/39 y | hospital | jaundice, nausea, vomiting,  diarrhea | micronodular cirrhosis, with fatty metamorphosis /  ethylic | none | DIC (laboratory verified) | 2,5-3 l nonclotted blood | 2000/  480/  480 | 127mg% | not mentioned | unknown | --- |
| DiMaio/  1987 [14] | F/38 y | hospital | none | none | none | yes | 1000 ml  unclotted blood | -/  -/  450 | negative | negative | unknown |  |
| DiMaio/  1987 † [14] | M/ late 50‘ | home | none | severe cirrhosis | --- | not mentioned | massive hemoperitoneum | --- | --- | --- | unknown | --- |
| Kim/  2007 [15] | F/34 y | home/sleeping | none | advanced micronodular cirrhosis, with fatty metamorphosis/ethylic | none | nasal bleeding for month, easily bruised | more than 3700 ml nonclotted blood + 50 ml clotted blood | 2527/  66/  287 | negative | acetaminophen, dextromerthophane, methylephe- drine, chlorpheniramine and caffein | unknown | --- |
| Kim/  2007  ‡ [15] | not mentioned | not mentioned | not mentioned | severe cirrhosis | --- | not mentioned | 720 ml clotted and nonclotted blood | --- | not mentioned | not mentioned | unknown | --- |
| Harbour/  2012 [16] | F/70y | hospital | nausea, weakness | not mentioned | hypertensive cardiovascular disease; general sever arteriosclerosis | none | 700 ml liquid and clotted blood | --- | not mentioned | not mentioned | arterial dissection of gastroduodenal artery | Mi |
| Harbour/  2012 [16] | F/ 55y | hospital | abdominal pain,  nausea,  diarrhea,  vomiting, dizziness | none | none | none | 5100 liquid and clotted blood (state after laparotomy) | --- | not mentioned | not mentioned | rupture of superior mesenteric and portal vein junction | --- |
| Dedouit  /2012 [13] | F/39y | home | none | cirrhosis/ethylic | chronic pancreatitis | none | 4 l liquid and clotted blood | 1700/  208/--- | 1,83 g/l | not mentioned | unknown | --- |
| Hayashi/  2013 [17] | M/43y | home | none | cirrhosis/ethylic | none | none | 3650 ml of blood | ---/  “enlarge”/--- | negative | negative | ectopic varices of mesentery of ascending colon (in postoperative adhesions) | Ma |
| Hayashi/  2013 [17] | F/66 y | home | none | cirrhosis (+ hepatocellular carcinoma) | none | none | 4 500 ml of fresh fluid blood | ---/  “enlarge”/--- | 0,2 mg/ml | negative | ectopic varices inguinal hernia sac of left labia majora | --- |
| Podduturi/  2014 [18] | F/59y | hospital | worsening dyspnea, confusion | cirrhosis (+ hepatocellular carcinoma) | asthma,  hypertension,  diabetes  mellitus | none | 5 900 ml of liquid and clotted blood | ---/---/--- | not mentioned | not mentioned | dissection of gastroduodenal artery | Mi |
| Battistini/ 2017 [19] | M/49 y | home | none | macronodular cirrhosis | none | none | 4650 ml liquid blood | 2160/  ---/  --- | negative | negative | unknown | --- |
| Battistini/ 2017 [19] | M/51 y | toilet in work | none | macro-/micro (mixed) nodular cirrhosis | none | none | 5100 ml liquid blood | 2120/---/  --- | negative | negative | unknown | --- |
| Kovařík/ 2018  [5] | M/31 y | hospital | symptoms of hypovolemic shock | steatocirrhosis | none | none | 6 liters of nonclotted bloody liquid | 3464/445/--- | 2,36 g/kg | toxic level of alprazolam | unknown | --- |
| Mileva/  2023 [20] | M/70‘ | home | none | none | generalized complicated atherosclerosis; signs of hypertensive and ischemic heart disease | none | cca 2,5—3 l liquid and clotted blood | --- | negative | negative | unknown | --- |
| Kovařík (*presented case*) | F/45 y | home | „felt sick“ | severe steatocirrhosis | DM II., arterial hypertension, mild chronic pancreatitis | hematuria, epistaxis | 3 liters of liquid and clotted blood | 1820/ 440/ 590 | negative | unimportant finding of mirtazapin, sertraline, carvedilol | subperitoneal ectopic varices of right diaphragm arch | Ma, Mi |

TABLE S1 Main features of all available forensic cases of ISIH. † – Case with failure to retain the autopsy report; ‡ – Case with incomplete description. (Abbreviations: F = female, M = male; DIC = disseminated intravascular coagulation; BAC = blood alcohol content; Ma = macroscopic image, Mi = microscopic image).
